# Rotavirus C Replication in Porcine Intestinal Enteroids Reveals Roles for Cellular Cholesterol and Sialic Acids

**DOI:** 10.3390/v14081825

**Published:** 2022-08-20

**Authors:** Yusheng Guo, Sergei Raev, Maryssa K. Kick, Molly Raque, Linda J. Saif, Anastasia N. Vlasova

**Affiliations:** 1 Center for Food Animal Health, Ohio Agricultural Research and Development Center, Department of Animal Sciences, College of Food, Agricultural and Environmental Sciences, The Ohio State University, Wooster, OH 44691, USA; 2 Department of Veterinary Preventive Medicine, College of Veterinary Medicine, The Ohio State University, Wooster, OH 44691, USA

**Keywords:** rotavirus C, porcine intestinal enteroids, cholesterol, sialic acids, histo-blood-group antigens

## Abstract

Rotaviruses (RVs) are a significant cause of severe diarrheal illness in infants and young animals, including pigs. Group C rotavirus (RVC) is an emerging pathogen increasingly reported in pigs and humans worldwide, and is currently recognized as the major cause of gastroenteritis in neonatal piglets that results in substantial economic losses to the pork industry. However, little is known about RVC pathogenesis due to the lack of a robust cell culture system, with the exception of the RVC Cowden strain. Here, we evaluated the permissiveness of porcine crypt-derived 3D and 2D intestinal enteroid (PIE) culture systems for RVC infection. Differentiated 3D and 2D PIEs were infected with porcine RVC (PRVC) Cowden G1P[1], PRVC104 G3P[18], and PRVC143 G6P[5] virulent strains, and the virus replication was measured by qRT-PCR. Our results demonstrated that all RVC strains replicated in 2D-PIEs poorly, while 3D-PIEs supported a higher level of replication, suggesting that RVC selectively infects terminally differentiated enterocytes, which were less abundant in the 2D vs. 3D PIE cultures. While cellular receptors for RVC are unknown, target cell surface carbohydrates, including histo-blood-group antigens (HBGAs) and sialic acids (SAs), are believed to play a role in cell attachment/entry. The evaluation of the selective binding of RVCs to different HBGAs revealed that PRVC Cowden G1P[1] replicated to the highest titers in the HBGA-A PIEs, while PRVC104 or PRVC143 achieved the highest titers in the HBGA-H PIEs. Further, contrasting outcomes were observed following sialidase treatment (resulting in terminal SA removal), which significantly enhanced Cowden and RVC143 replication, but inhibited the growth of PRVC104. These observations suggest that different RVC strains may recognize terminal (PRVC104) as well as internal (Cowden and RVC143) SAs on gangliosides. Finally, several cell culture additives, such as diethylaminoethyl (DEAE)-dextran, cholesterol, and bile extract, were tested to establish if they could enhance RVC replication. We observed that only DEAE-dextran significantly enhanced RVC attachment, but it had no effect on RVC replication. Additionally, the depletion of cellular cholesterol by MβCD inhibited Cowden replication, while the restoration of the cellular cholesterol partially reversed the MβCD effects. These results suggest that cellular cholesterol plays an important role in the replication of the PRVC strain tested. Overall, our study has established a novel robust and physiologically relevant system to investigate RVC pathogenesis. We also generated novel, experimentally derived evidence regarding the role of host glycans, DEAE, and cholesterol in RVC replication, which is critical for the development of control strategies.

## 1. Introduction

Rotaviruses (RVs) are a significant cause of severe diarrheal illness in infants and young animals, including pigs [1]. In children, RV infections are associated with approximately USD 96 million in annual hospitalization-related costs in the United States alone [2,3]. 

RVs are non-enveloped double-stranded RNA (dsRNA) viruses that contain 11 segments of dsRNA encoding for structural and non-structural proteins: VP1-VP4, VP6, VP7, and NSP1-5/6. RVs are classified into nine antigenically and genetically distinct groups according to the International Committee on the Taxonomy of Viruses (ICTV, https://talk.ictvonline.org/taxonomy/, accessed on 16 August 2022), designated as RVA, RVB, RVC, RVD, RVF, RVG, RVH, RVI, and RVJ [4,5,6,7]. Within each group, RVs are further classified into G and P genotypes based on the molecular characteristics of their outer surface glycoprotein VP7 and the protease-sensitive spike protein VP4 (cleaved into VP8* and VP5* under protease treatment), respectively [8,9]. RVA has been studied extensively and historically considered the most prevalent and pathogenic among the nine RV groups [9].

RVCs are emerging pathogens increasingly reported in pigs and humans worldwide and are currently recognized as the major cause of gastroenteritis in neonatal piglets [10,11,12,13]. RVCs have been documented as single or mixed infections with other enteric pathogens in nursing, weaning, and post-weaning pigs [10,14,15,16]. Single RVC infections are being increasingly identified in association with diarrhea in very young suckling piglets, and higher RVC RNA titers are significantly associated with diarrhea in piglets [17,18]. Moreover, seroepidemiological studies in pigs indicated that RVCs have been circulating in swine herds in different countries for many decades, reaching seroprevalence rates of 58–100% [10]. Recent studies demonstrated very high prevalence and significant genetic diversity of porcine RVCs in different countries [13,17,19,20,21]. Despite the increasing importance of RVCs, our knowledge of RVC pathogenesis is still limited due to the lack of a robust cell culture system for most RVC strains and only a few studies focusing on RVC pathogenesis. Notwithstanding significant efforts made to develop a suitable in vitro culture system, the porcine RVC Cowden strain was the first of only a few cultivable RVCs that can be serially propagated in porcine kidney cell cultures and are characterized by modest replication titers [22]. Thus, the development of a suitable and robust cell culture system is essential to understand the basic virology, pathogenesis, and innate immune and other cellular responses induced by RVCs.

Intestinal enteroids (IEs) are in vitro, three-dimensional (3D) culture systems that recapitulate the complex nature and functions of the gut [23,24,25,26]. IEs are isolated from epithelial stem cells residing in small intestinal crypts and cultured in Wnt3A-rich growth medium and Matrigel, which support their 3D structure [24]. The crypt cells give rise to stem and transient amplifying cells, and all of the small intestinal differentiated epithelial cell lineages, including enterocytes, enteroendocrine cells, goblet cells, and Paneth cells, identified in humans, mice, and pigs [25,27,28]. Tuft cells, which represent approximately 1% of the total epithelial cell population, are also reported to exist in IEs [29]. IEs have recently demonstrated tremendous potential as a model to study pathogen–host interactions, including norovirus, porcine epidemic diarrhea virus (PEDV), porcine deltacoronavirus (PDCoV), severe acute respiratory syndrome coronavirus 2, and RV [27,28,30,31,32,33]. Importantly, IEs express glycan receptors, such as sialic acids (SAs), and histo-blood-group antigens (HBGAs), which makes them an indispensable in vitro model to investigate the host glycan–RV interactions and dissect the mechanisms of RV cell entry [7,34].

Receptors or attachment factors associated with RVC attachment and internalization have not been identified yet. Limited studies have indicated that SAs and HBGAs may play a role in RVCs’ cell entry. For example, a previous study revealed that the RVC Amc-1 strain required SAs for erythrocyte and cell-receptor binding [35]. A recent study by Sun and colleagues demonstrated that VP8* of human RVC G4P [2] recognizes type A HBGAs through the use of glycan microarrays [36]. Similarly, another study indicated that VP8* of two human RVC strains recognized type A HBGA, and VP8* of a bovine RVC strain could bind to a tri-saccharide glycan Galα1-3Galβ1-4Glc that contained a terminal α-Gal [37]. Additionally, cell culture additives, such as diethylaminoethyl (DEAE)-dextran, cholesterol, and bile acids were reported to improve the replication of various RVs [30,38,39,40]. Whether these additives play a significant role in RVC cell entry and replication remains to be established.

In this study, we tested if the porcine intestinal enteroid (PIE) culture system established previously in our lab [7] is permissive to RVC infection and identified the optimal culture conditions for three porcine RVC (PRVC) strains of different genotypes, including PRVC Cowden G1P[1], PRVC104 G3P[18], and PRVC143 G6P[5] [41]. We also examined if RVC cell entry and replication were SA- or HBGA-dependent. Finally, we tested whether cell culture additives, such as DEAE-dextran, cholesterol, and bile extract, could affect the replication of PRVC.

## 2. Materials and Methods

### 2.1. Rotavirus C Strains

Gnotobiotic pig intestinal contents containing RV104 G3P[18] and RV143 G6P[5] and Cowden G1P[1] PRVC strains were used in this study. The contents were diluted 1:10 in sterile Minimal Essential Medium (MEM Gibco; Life Technologies, Grand Island, NY, United States), clarified by centrifugation at 2000× *g* for 15 min at 4 °C, and filtered through a 0.2 µm filter as described [41,42]. Immediately prior to PIE infection, all the virus pools were adjusted to the desired titer.

### 2.2. Three-Dimensional Enteroid Propagation, Passaging, and Differentiation

Crypt cells were extracted from the small intestine (ileum) as described previously [24], with slight modifications detailed in Guo et al. [7]. The culture conditions for porcine IEs’ (PIEs’) maintenance and differentiation were formulated as follows: complete medium without growth factors (CMGF(-) medium) consisted of advanced DMEM/F12 medium (Invitrogen, US) supplemented with 100 U/mL penicillin–streptomycin (PS, Invitrogen), 10 mM HEPES buffer (Invitrogen), and 1% of GlutaMAX-100x (Invitrogen) [24,28]. The complete cell culture medium contained IntestiCult Organoid Growth Medium (Component A: Component B ratio = 1:1) (STEMCELL), 100 U/mL of PS (Invitrogen), and 2.5 µM of CHIR99021 (Stemgent). The differentiation medium (DM) contained IntestiCult Organoid Growth Medium Components A and B added at a ratio of 1:0.8, supplemented with 100 U/mL of PS (Invitrogen). 

### 2.3. Two-Dimensional Enteroid Monolayer Establishment, Maintenance, and Differentiation

We used previously established protocols for growing PIEs as monolayers [43], with some modifications. Briefly, culture wares were coated with diluted Matrigel^®^ solution. An aliquot of Corning^®^ Matrigel^®^ was thawed and kept on ice until use. An appropriate amount of cold (2–8 °C) Dulbecco’s phosphate-buffered saline (DPBS) was dispensed at 100 µL/well in 96-well plates. Then, thawed Matrigel^®^ was added to the cold DPBS (1 μL of Matrigel^®^ to 49 μL DPBS), mixed well, and kept on ice. The diluted Matrigel^®^ solution was used immediately to coat the tissue culture-treated culture wares. Then, the culture plates were incubated at 37 °C for 1 h before use. The excess Matrigel^®^ solution was removed using a serological pipette ensuring that the coated surface was not scratched. 

Two-dimensional monolayer growth medium (2D-MM) was prepared as follows (example is for preparing 120 mL of monolayer growth medium): 50 mL of IntestiCult™ Component A + 50 mL of IntestiCult™ Component B + 100 µL Y-27632 + 25 µL CHIR99021 + 20 mL fetal bovine serum (FBS, Invitrogen, US). The desired amount of antibiotics was added immediately before use. 

For plating PIEs as a monolayer, the seeding density of PIEs in 96-well plates was 1.0–2.5 × 10^4^/well. The cell numbers of 3D PIE generally reached ~1–2 × 10^5^ cells/dome. Sufficient numbers of domes were harvested to achieve the desired seeding density. One mL of gentle cell-dissociation reagent (GCDR, STEMCELL, Canada) was added to each well containing a dome to be harvested; then, the domes were incubated at room temperature (20–25 °C) for 1 min, followed by vigorous pipetting using a 1000 μL pipettor to disrupt the Matrigel^®^ dome. The fragmented PIEs were then incubated at room temperature for 10 min with gentle rocking. Then, the harvested PIE fragments were pooled and centrifuged at 400× *g* for 5 min at 4 °C. The supernatant was discarded and 3 mL of ice-cold CMGF- was added, followed by vortexing and centrifuging at 400× *g* for 5 min at 4 °C. This step was repeated twice. Then, the supernatant was gently removed, followed by resuspending the PIE fragments in 1 mL of warm (37 °C) trypsin-ethylenediaminetetraacetic acid (trypsin-EDTA, 0.05%), vigorous vortexing, and incubation at 37 °C for 10 min. Following that, 3 mL of CMGF- supplemented with 10% FBS was added and mixed thoroughly by pipetting, and centrifuged at 900× *g* for 5 min at 4 °C. Then, the supernatant was removed, and the PIE fragments were resuspended in 1 mL of the 2D-MM, as indicated above. The PIE fragments were then passed through a 25 G needle multiple times to disrupt their structure to achieve single-cell suspensions. After cell counting and adjustment to the desired concentration, the cell suspension was slowly and gently added to each well and incubated at 37 °C under 5% CO_2_, and the medium was replaced every 2–3 days until monolayers were formed. If differentiated PIEs were desired, the medium was changed from 2D-MM to DM two days after seeding, and the monolayers were incubated in it for two days.

### 2.4. Immunofluorescence (IF)

The IF staining of 3D PIEs was conducted as described previously [7]. The IF staining of 2D PIEs was conducted as follows. Poly-lysine-treated cover glass slides were used for the staining of 2D PIEs cultures. Briefly, the cover glass was coated with an appropriate amount of poly-lysine for 1 h at room temperature, followed by rinsing the coverslips with sterile H_2_O (three times for 1 h each). Then, the coverslips were air-dried and sterilized under UV light for at least 4 h. One cover glass was added to each well of 6-well cell culture plates, coated with 1 mL of diluted Matrigel, and 2D PIEs were grown on glass coverslips as described above. On the day of staining, the 2D culture medium was removed from the plate and washed twice with phosphate-buffered saline (PBS). Then, 1 mL of 4% paraformaldehyde in PBS (pH 7.4) was added, incubated for 10 min at room temperature, and washed three times with PBS, followed by antigen retrieval steps. Antigen retrieval, permeabilization, blocking, and antibody staining were conducted as described previously [7]. The following primary and secondary antibodies were used for staining: rabbit SOX9 (1:100, Millipore, US), goat anti-human villin C-19 (1:100, Santa Cruz, US), mouse anti-RVC (1:50, Kerafast, US), Alexa Fluor 488 goat anti-rabbit IgG (1:500, Thermo Fisher Scientific, US), Alexa Fluor 488 rabbit anti-goat IgG (1:500, Thermo Fisher Scientific, US), and Alexa Fluor 488 goat anti-mouse IgG (1:100, Thermo Fisher Scientific, US). Slides were sealed by adding the mounting medium, and images were captured using a Keyence BZ-810 microscope.

### 2.5. RV Inoculation of PIEs

We used the protocol described previously with slight modifications [7]. Briefly, to achieve the correct multiplicity of infection (MOI), 1 mL of Accutase cell-dissociation solution (BD Biosciences, US) was added to each well with PIEs and incubated at 37 °C for 30 min. The MOI was calculated as the amount of the input virus divided by the total number of cells in the samples (dissociated PIEs) [28]. The cell concentration was determined using a Cellometer Auto T4 (Nexcelom Bioscience, US). For RV infection, the PIEs were differentiated in DM for at least 4 days. An amount of 500 µL of GCDR was used to remove Matrigel, and the PIE fragments were apically exposed to RVCs as described previously [28]. The RVC inocula were pre-activated using 10 µg/mL trypsin for 30 min at 37 °C and diluted with the loading solution containing CMGF- and 10 µg/mL trypsin to achieve the desired MOI (set at 1 unless specified otherwise). PIEs were incubated with the RVC inocula for 2 h at 37 °C, and then washed twice using a cold CMGF- medium and resuspended in an appropriate amount of loading solution. An amount of 100 µL of the RVC-exposed PIEs was plated in 96-well plates in triplicate, and the plates were harvested at specific time points and frozen at −80 °C until use. The cell concentration was determined using a Cellometer Auto T4 (Nexcelom Bioscience, US). 

For 2D infection, the conditional medium from differentiated PIEs was removed, CMGF- was added, and plates were incubated for 3 h at 37 °C. The RVC inocula were pre-activated with 10 µg/mL trypsin at 37 °C for 30 min. The loading solution was prepared by adding pancreatin (Invitrogen) to CMGF- at a final concentration of 0.25 mg/mL. The virus stock was then diluted (using loading solution) to 1 × 10^4^ FFU/mL to achieve an MOI of 0.1. After that, we added 50 µL of loading solution and 50 µL of virus inoculum into the wells of 96-well plates and incubated them at 37 degrees for 2 h. The virus inoculum was then removed, and the plates were washed twice using CMGF- medium. Fresh CMGF- was added to the plates, the plates were placed in an incubator at 37 °C, and the virus was harvested at 2 and 48 h post-infection.

### 2.6. 2F-Peracetyl-Fucose and Sialidase Treatment

Briefly, 2F-peracetyl-fucose (2F) is a cell-permeable fluorinated fucose derivative that acts as an inhibitor of fucosyltransferases following its uptake and metabolic transformation into a GDP-fucose mimetic [44,45,46]. We used the protocols described previously [7]. For 2F (Sigma-Aldrich, US) treatment, 500 μM of 2F or dimethyl sulfoxide (DMSO, negative control) was added to differentiated PIEs and incubated for 3 days at 37 °C before they were infected with RVC as described above (fresh 2F/DMSO were added daily). Cell viability was examined using Trypan blue before infection to ensure no DMSO-associated toxic or other biological effects on PIEs. The infected PIEs were incubated for 2 days before harvesting. For sialidase treatment, MA104 cells in 96-well plates or PIEs were pre-treated with 10 mU (diluted in 50 mM Tris-HCl pH 7.5, 150 mM NaCl, 10 mM CaCl_2_, and 0.02% NaN_3_: TNC buffer) of sialidase/neuraminidase (NA) from *Arthrobacter ureafaciens* (Roche, US) for 1 h at 37 °C before inoculation. TNC buffer was used as a negative control for sialidase treatment. Following sialidase treatment, the PIEs were infected with RVCs and harvested as described above.

### 2.7. Cell Culture Additives, Cholesterol-Depletion Assay, and Cholesterol Level Measuring

DEAE-dextran (Sigma, US), porcine bile extract (Sigma, US), and water-soluble cholesterol (Sigma, US) dissolved in DPBS or Milli-Q water (cholesterol only) were added to PIEs to achieve final concentrations of 50 µg/mL, 20 µg/mL, and 10 µg/mL, respectively. CMGF- containing the above additives or CMGF- alone (used as a negative control) were removed following 1 h of incubation at 37 °C, and the PIEs were washed twice with CMGF- medium and then infected with RVCs as described above. 

Methyl-β-cyclodextrin (MβCD, Sigma, US), which has a high affinity for inclusion in the cell wall, competing with cholesterol and causing its cellular depletion, was used in the cholesterol-depletion assay. The cholesterol-depletion protocol has been described previously, and we used it with slight modifications [39]. Briefly, differentiated 3D PIEs were treated with 0 mM, 5 mM, or 20 mM MβCD at 37 °C for 1 h. For cholesterol replenishment, the PIEs were pretreated with 20 mM MβCD at 37 °C for 1 h, followed by washing with CMGF-, and then the cells were incubated with 1mg/mL (10 µg in total) of exogenous cholesterol for 1 h at 37 °C. To measure the PIE cholesterol levels, an Amplex™ Red Cholesterol Assay Kit (Invitrogen™) was used following the manufacturer’s instructions.

### 2.8. RNA/DNA Extraction and PCR/qRT-PCR

As described previously [7], lysis buffer containing 0.5 mg/mL protease K (Amresco, US), 50 mM KCl (Sigma), 10 mM Tris-Cl, pH 8.0 (Invitrogen), 2.5 mM MgCl_2_ (Sigma), 0.45% IGEPAL (Sigma), 0.45% Tween-20 (Promega, US), and DEPC-treated water (Promega) was used for the total RNA extraction. Briefly, 200 µL of lysis buffer was added to each well of 96-well plates that contained PIEs and was incubated for 30 min at 37 °C. Then, 100 µL of the PIE–lysis buffer mixture was transferred to a 96-well PCR plate, followed by incubation in a PCR cycler with the following parameters: 30 min at 65 °C and 2 min at 98 °C, and the samples were then cooled down to 4 °C. The RNA concentration was measured by NanoDrop2000c and adjusted to the same level. A Qiagen one-step RT-PCR kit was used for RVC detection using the primers, probe, and protocol described previously [18]. The Ct values from the RT-PCR were converted to FFU/mL based on a standard curve generated using the infectious titer of the cell culture-adapted RVC Cowden. Our calculations demonstrated that the mRNA quantities correlated with the CCIF (infectious virus titers) results (R^2^ = 0.98).

### 2.9. Statistical Analysis

GraphPad Prism v 5.0 (GraphPad Software, San Diego, CA, USA) was used for data analysis. Comparison of the viral titers between different treatments was done using Student’s *t*-test unless specified. Differences were considered statistically significant when *p*  ≤  0.05.

## 3. Results

### 3.1. RVCs Replicate More Efficiently in 3D-PIEs Expressing Certain HBGAs, but the Replication Is Independent of the Presence of HBGAs

**Figure 1:**
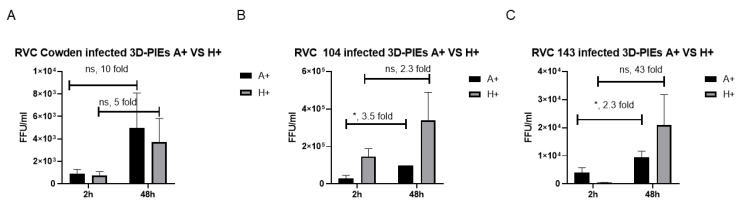
RVC replication in HBGA-A+ and HBGA-H+ PIEs. RT-PCR infectivity in A+ (black), H+ (gray) for (**A**) PRVC Cowden, (**B**) PRVC104, and (**C**) PRVC143. RVC titers were examined at 2 h and 48 h. Error bars are denoted as the standard deviation, and all experiments were repeated independently at least twice. Ns denotes no significant difference. Capped lines indicate the fold-changes between two groups. For *p*-value: * *p* < 0.05.

In this study, we first evaluated whether 3D-PIEs support RVC replication and if PIEs expressing different HBGA types support RVC replication to different levels. To evaluate the replication efficiency of RVCs in PIEs expressing different HBGA types (A+ and H+, the major HBGAs antigens in swine) [47], differentiated PIEs were inoculated with the three PRVC strains (Cowden, RVC104, and RVC143), and the RVC mRNA levels were measured by qRT-PCR at 2 h post-inoculation (h) and 48 h. The results indicated that Cowden reached the highest titer in A+ PIEs, while PRVC104 and PRVC143 replicated better in H+ PIEs at 48 h (Figure 1A–C), suggestive of strain-specific affinity for individual HBGAs, as we observed for RVAs [7].

**Figure 2:**
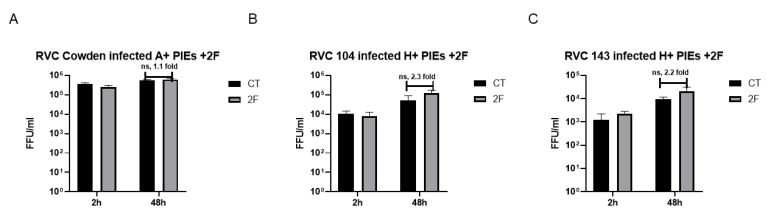
RVC replication in PIEs with or without 2F treatment. An amount of 500 μM 2F or CT (DMSO) was added to differentiate HBGA-A+ and HBGA-H+ PIEs for 3 days (media with 2F/DMSO were refreshed every day), and then infect them with RVC strains, followed by incubation for 2 days. Then, 2F-treated PIEs were used for RT-PCR detection of (**A**) PRVC Cowden, (**B**) PRVC104, and (**C**) PRVC143 in A+, H+, and H+ PIEs, respectively. RVC titers were examined at 2 h and 48 h. The error bars represent the standard deviation from triplicate samples. All experiments were repeated independently at least 2 times. ns denotes no significant difference. Capped lines indicate the fold-changes between two groups.

To further confirm the role of HBGAs in RVC infection, a fucosyltransferase inhibitor, 2F [44,45,46], was used to inhibit the expression of HBGAs in the newly synthesized PIEs. To test whether absence or limited HBGA expression inhibits RVC infection, 2F-treated A+ PIEs were inoculated with RVC Cowden, and 2F-treated H+ PIEs were inoculated with PRVC104 and PRVC143 strains. The results demonstrated no significant differences in RVC replication in the 2F-treated or the control (DMSO treated) PIEs at 48 h (Figure 2A–C). Together, these results indicate that HBGA specificity may affect RVC replication in 3D PIEs, but the infection is not dependent on the presence of HBGAs.

### 3.2. Terminal SAs May Play Contrasting Roles in RVCs Infection

**Figure 3:**
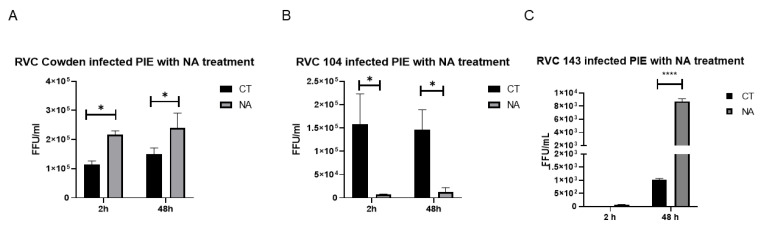
RVC replication in PIEs with or without sialidase treatment. Replication of virulent (**A**) Cowden, (**B**) PRVC104, and (**C**) PRVC143 after PIEs’ sialidase treatment, respectively. Differentiated PIEs were pre-treated with 10 mU sialidase (NA) from *Arthrobacter ureafaciens* or CT (TNC buffer) for 1 h at 37 °C before inoculation. Then, PIEs were inoculated with 10^5^ FFU of RVCs and incubated at 37 °C. PIEs were harvested at 2 h and 48 h, and the virus titers were measured by RT-PCR. The error bars represent the standard deviation from triplicate samples. All infectivity assays were repeated independently at least twice. For *p*-value: * *p* < 0.05, **** *p* < 0.0001.

Because we observed no inhibitory effects of the 2F treatment on RVC replication, we wanted to further explore the role of another receptor—Sas—in RVC attachment/entry. The replication of Cowden, PRVC104, and PRVC143 was evaluated in PIEs that were pre-treated with 10 mU of NA or TNC buffer (control, CT). Our results demonstrated that the replication of PRVC104 was significantly decreased following NA treatment, suggesting the sialidase-sensitive nature of the RV104 strain (Figure 3B). In contrast, the replication of Cowden and PRVC143 was greatly enhanced in the NA-treated PIEs (Figure 3A,C, respectively), similar to our previous observations of RVA G9P[13] [7]. These data indicate that internal Sas or other unknown receptors may remain masked by terminal Sas in the absence of NA treatment. Together, these experiments highlight the contrasting mechanisms of interactions between Sas and PRVC104 vs. Cowden/PRVC143 strains.

### 3.3. DEAE-Dextran Significantly Enhanced RVC Adsorption on the Host Cells

**Figure 4:**
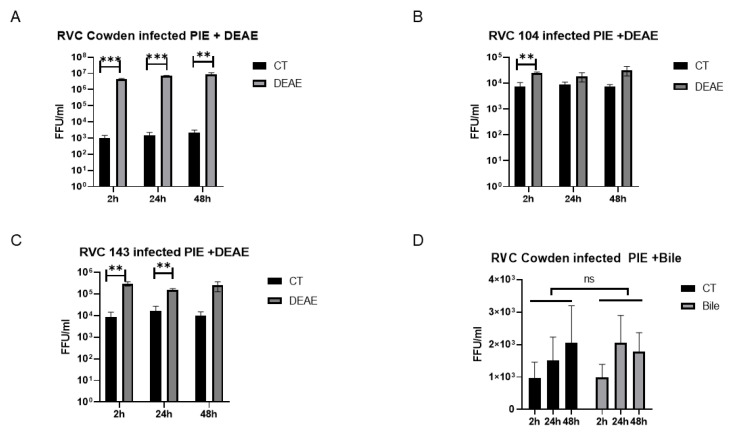
Effect of DEAE and bile extract on RVC replication in PIEs. Replication of (**A**) Cowden, (**B**) PRVC104, and (**C**) PRVC143 in DEAE-dextran-treated PIEs, respectively. DEAE was added to virus inocula to a final concertation of 50 µg/mL. (**D**) Cowden-infected PIEs with bile extraction. Porcine bile extraction was added to virus inoculum to a final concertation of 20 µg/mL. Plates were harvested at 2 h, 24 h, and 48 h, and the virus titers were measured. The error bars represent the standard deviation from triplicate samples. The experiments were repeated independently at least twice. For *p*-value: ** *p* < 0.01, and *** *p* < 0.001. ns denotes no significant difference. Group difference analysis in D was measured by a one-way ANOVA.

Cell culture additives, such as DEAE-dextran, bile acids, and cholesterol, were previously reported to increase the propagation of RVs and other viruses [30,38,39,40]. Thus, we wanted to test whether these supplements could also enhance RVC replication in PIEs. The first additive we used was DEAE-dextran, a positively charged dextran derivative that can be used for the transfection of foreign DNAs. We observed that 50 µg/mL of DEAE-dextran significantly enhanced the amount of virus adsorbed on PIEs at 2 h for all RVC strains evaluated compared with the DPBS control (Figure 4A–C). However, subsequently, the virus titer of the Cowden strain was only slightly increased by 24 h and 48 h in DEAE treated group (Figure 4A), whereas the titers of PRVC104 and PRVC143 maintained similar levels during the experiment (Figure 4B,C). These data indicate that DEAE-dextran may affect RVC attachment, but not internalization, as it had no effect on RVC replication titers. We also tested if the addition of swine bile extract affected RVCs infection; however, we did not observe any significant difference between the bile-treated and control (DPBS) groups (Figure 4D).

### 3.4. Cowden Infection of PIEs Is Dependent on Cellular Cholesterol

**Figure 5:**
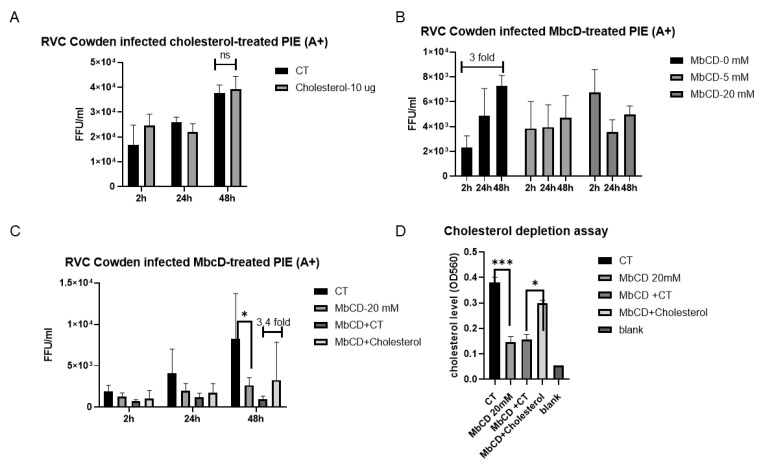
Cholesterol depletion and replenishment effects on Cowden RVC replication in PIEs (A+) (**A**) Treatment of PIEs with exogenous cholesterol. PIEs were either incubated with 10 µg cholesterol or CT (water) for 1 h at 37 °C before virus inoculation. (**B**) Treatment of PIEs with MβCD (cholesterol depletion). PIEs were treated with 0 mM (water), 5 mM, and 20 mM MβCD at 37 °C for 1 h before virus inoculation, respectively. (**C**) Cholesterol replenishment of PIEs. PIEs were incubated with water control (CT), MβCD, MβCD without cholesterol replenishment (MβCD + water), and MβCD with cholesterol replenishment (MβCD + Cholesterol). For cholesterol replenishment, PIEs pretreated with MβCD were washed with CMGF-; then, the cells were incubated with 1 mg/mL of exogenous cholesterol for 1 h at 37 °C. (**D**) Quantification of PIEs’ cellular cholesterol level. An Amplex™ Red Cholesterol Assay Kit (Invitrogen™) was applied following the manufacturer’s instructions. The cholesterol level is a positive related value of OD560. After virus inoculation, the plates were harvested at 2 h, 24 h, and 48 h, and the virus titers were measured. The error bars represent the standard deviation from triplicate samples. All infectivity assays were repeated independently at least twice. For *p*-value: * *p* < 0.05, and *** *p* < 0.001. ns denotes no significant difference. Capped lines indicate the fold-changes between two groups.

Many studies have demonstrated that cellular cholesterol is required for multiple viruses, such as porcine reproductive and respiratory syndrome virus, transmissible gastroenteritis virus, bovine herpesvirus type 1, bovine RV, murine leukemia virus, and simian virus 40 [39,48,49,50,51,52]. To explore how exogenous and cellular cholesterol affect RVC infection, we designed a series of experiments. In the first, we added 10 µg of exogenous cholesterol to treat the PIEs before inoculating them with the PRVC Cowden strain. A slight increase (not significant) in the virus titer was observed at 2 h, but not at 24 h and 48 h, in the cholesterol-treated groups compared with the control (CT) group (Figure 5A). In the next experiment, MβCD was applied to deplete the cellular cholesterol, followed by Cowden inoculation. Our results indicated that, starting at 5 mM, MβCD was able to inhibit the replication of Cowden (Figure 5B), suggesting that cellular cholesterol may be required for RVCs infection. To further confirm the role of cholesterol, a cholesterol-depletion and restoration assay was performed. Briefly, four groups of PIEs were inoculated with Cowden, including CT, MβCD single treatment (MβCD), MβCD without cholesterol (MβCD + water), and MβCD with cholesterol (MβCD + cholesterol). We observed that the cellular cholesterol level was decreased in the MβCD group compared with the CT group. The supplementation of exogenous cholesterol (EC) restored its levels to 75% of the CT group (Figure 5D). The qRT-PCR results revealed that MβCD treatment significantly inhibited Cowden growth at 48 h, while supplementation with cholesterol (MβCD + Cholesterol) partially rescued the inhibitory effect of MβCD compared with the MβCD + water group. Interestingly, the cholesterol level resulting from EC supplementation restored did not correlate linearly with the increase in the virus titer. Nevertheless, these results indicated that RVC replication in PIEs is dependent on cellular cholesterol levels.

### 3.5. RVCs Replicated Well in 3D-PIEs but Replicated Poorly in 2D-PIEs

**Figure 6:**
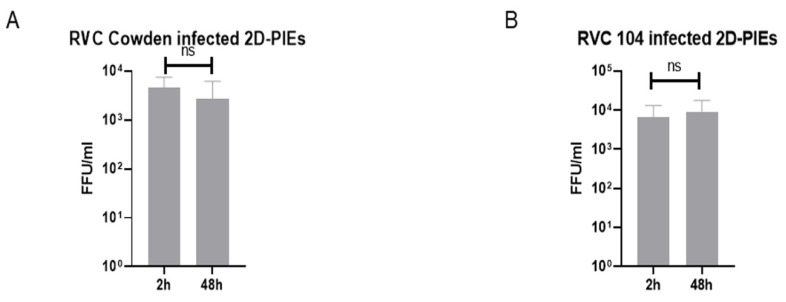
RVC infection of 2D-PIEs. (**A**) Cowden and (**B**) PRVC104-infected 2D-PIE. RV load was examined at 2 h and 24 h. Error bars indicate the standard deviation, and all experiments are 2–3 independent replicates. Capped lines indicate the fold-changes between two groups. ns denotes no significant difference.

Recently, 3D-back-to-2D enteroid monolayer cultures were established and found to recapitulate many of the features of 3D cultures [53]. Although convenient, the utilization of PIE monolayers vs. 3D cultures needs to be considered carefully for each specific virus. In our previous study, RVAs replicated well in 3D-PIEs [7], and we confirmed that RVCs also replicated efficiently in 3D-PIEs (Figure 1). Thus, we wanted to determine if 2D-PIEs support RVCs replication. In contrast to 3D-PIEs, RVCs strains replicated poorly in 2D-PIEs (Figure 6A,B), with modest titer increases by 24 h post-infection (non-significant, ns). Thus, we concluded that 2D-PIEs marginally supported RVC infection and replication.

To explore why 2D-PIEs failed to support RVC infection, we compared the expression of SOX9 (stem cell marker) and villin (enterocyte marker) in 3D cultures vs. 2D monolayers by IF (Figure S1; Table 1). Our results revealed that higher numbers of stem cells (56.5% vs. 34.6% of the total cell number) and lower numbers of mature enterocytes (24.3% vs. 85.5% of the total cell number) were present in the 2D-PIEs monolayers vs. 3D-PIEs. These results suggest that RVC infection may require a higher number of terminally differentiated enterocytes, as also suggested by the RVC antigen distribution in enterocytes at the terminal portion of the villi from in vivo pathogenesis studies [54].

## 4. Discussion

Robust in vitro models are prerequisites for studies of molecular mechanisms associated with virus cell entry, replication, and the pathogenesis of many endemic and emerging viruses. As it threatens the pork industry and public health, RVC represents an important virus for further studies. However, our knowledge of RVC entry and host/tissue restriction factors is limited due to the lack of a suitable cell culture system. Several studies have reported successful serial passaging for some of the RVC strains in several cell lines, including MA104 [42,55] and swine testicular cells [56]. However, these cell lines failed to support the robust replication of other field RVC strains, and even the replication of the cell-culture-adapted RVC strains remains challenging. In this study, our 3D-PIE model supported the robust replication of several RVC strains, including Cowden, RVC104, and RVC143. 

One study suggested that transition from 3D PIEs to 2D monolayers may alter the relative expression levels of different epithelial cell markers, indicating a decrease in goblet and Paneth cells in the monolayers [57]. Our data revealed similar findings (Table 1). The cell characteristics of 3D and 2D-PIEs could be different, including variable interactions between the cellular and extracellular environments, changes in cell morphology, and polarity. These differences could have contributed to the observed limited replication of RVCs in the 2D PIEs.

HBGAs [58,59,60,61,62] and SAs [63,64,65,66,67,68] have been proposed to be the receptors or attachment co-factors of RVA. Limited studies indicated that SAs and HBGAs may play a role in RVC cell entry [35,36,37], but the role of HBGAs and SAs in RVC infection is not fully understood. Our current results demonstrating the variable efficacy of replication in A+ vs. H+ PIEs and the ability to replicate in 2F-treated PIEs suggested that, while HBGAs serve as important cofactors for virus attachment/entry, they are not the only cellular receptors for RVC binding. We also confirmed the importance of terminal SA for RVC attachment/entry and demonstrated that RVC104 is a sialidase-sensitive strain (Figure 3B). In contrast, Cowden and RVC143 were demonstrated to be NA-dependent strains, where NA treatment enhanced their replication (Figure 3A and Figure 3C, respectively) which is similar to recent findings for G9P[13] [7]. These data suggest some unknown factors masked by terminal SA may be essential for the attachment and replication of some RVA and RVC strains [68]. Glycans that contain internal SA (gangliosides for instance) may be a potential target to investigate the mechanisms of the enhanced replication of RVs after sialidase treatment. We recently checked the amount of internal SAs GM1 on the cell surface with/without NA treatment, and demonstrated that GM1 (the prototype ganglioside) accessibility was improved following NA treatment (data not shown). Further, GM1 blocking by its ligand cholera toxin subunit B inhibited G9P[13] replication in NA-treated MA104 cells, indicating that GM1 may represent an alternative attachment factor during G9P[13] infection [69]. Whether GM1 also interacts with the Cowden strain remains to be explored. In summary, HBGAs may serve as a co-factor, while SAs may play a more important role in RVC cell attachment and entry.

Cell culture supplements and additives compensate for characteristics that the basal medium lacks and provide optimal cell-growth conditions. Our current results suggest that DEAE-dextran may affect RVC attachment, but not replication. Earlier studies indicated that the presence of polycations enhanced absorption, but also interfered with the release of reovirus [70]. This may explain our current observations demonstrating that RVC titers peaked at 2 h, but failed to increase from 24 h to 48 h in DEAE-treated PIEs. Nonetheless, the attachment and internalization process of RVC were not specifically investigated, and the mechanism of how polycations assist in RVC infection requires further investigation.

Although the addition of external cholesterol did not seem to affect RVC replication, the physiological levels of cellular cholesterol seemed to be essential for the efficient infection of RVC Cowden. More importantly, replenishing the cellular cholesterol levels partially rescued the titer decrease caused by MβCD. These findings suggest that cellular cholesterol plays an important role in RVC infection. As an essential part of the lipid raft membrane, cellular cholesterol is associated with various physiological processes, such as lipid sorting, protein trafficking, cell polarization, and signal transduction [71,72,73]. Cholesterol-enriched membrane microdomains have also been reported to interact with multiple stages of the virus life cycle, including entry, fusion, replication, assembly, and budding [74,75,76]. Thus, it is reasonable to hypothesize that cellular cholesterol may affect RVC infection through the cholesterol-enriched membrane microdomains. Additionally, it is interesting that the effect of the exogenous cholesterol could not completely rescue viral replication. Possible explanations for this observation could be: (1) exogenous cholesterol being inefficiently taken up by the 3D PIE cultures or (2) different incubation times of external cholesterol affecting the rescue effect of the cholesterol. Nonetheless, further studies are required to confirm the molecular mechanisms.

Our study provides the first evidence that several virulent RVC strains can be efficiently propagated in the 3D-PIEs culture. The 2F treatment and NA treatment assays revealed that HBGAs may serve as co-factors, while SAs could play an important role in RVC infection. Finally, our experiments confirmed that, similar to RVAs, cellular cholesterol is one of the essential factors for RVC replication. Overall, our study has generated a robust tool to investigate RVC pathogenesis and has broadened our knowledge regarding the roles of SAs and cholesterol in RVC replication, which may be important for the development of preventative and interventional strategies currently unavailable for RVCs.

## Figures and Tables

**Table 1:** Cell population comparison between 3D and 2D-PIEs.

	3D-PIEs	2D-PIEs
SOX9+ (stem cells)	34.6%	56.5%
Villin (enterocytes)	85.5%	24.3%

## Data Availability

Not applicable.

## Supplementary Materials

The following supporting information can be downloaded at: https://www.mdpi.com/article/10.3390/v14081825/s1, Figure S1: Maker expression of the 2D monolayer.

## References

[1] Tate J.E., Burton A.H., Boschi-Pinto C., Parashar U.D., World Health Organization-Coordinated Global Rotavirus Surveillance Network (2016). Global, regional, and national estimates of rotavirus mortality in children <5 years of age, 2000–2013. Clin. Infect. Dis..

[2] Parashar U.D., Gibson C.J., Bresee J.S., Glass R.I. (2006). Rotavirus and severe childhood diarrhea. Emerg. Infect. Dis..

[3] Kilgore A., Donauer S., Edwards K.M., Weinberg G.A., Payne D.C., Szilagyi P.G., Rice M., Cassedy A., Ortega-Sanchez I.R., Parashar U.D. (2013). Rotavirus-associated hospitalization and emergency department costs and rotavirus vaccine program impact. Vaccine.

[4] Matthijnssens J., Ciarlet M., Heiman E., Arijs I., Delbeke T., McDonald S.M., Palombo E.A., Iturriza-Gómara M., Maes P., Patton J.T. (2008). Full genome-based classification of rotaviruses reveals a common origin between human wa-like and porcine rotavirus strains and human ds-1-like and bovine rotavirus strains. J. Virol..

[5] Chen F., Knutson T.P., Porter R.E., Ciarlet M., Mor S.K., Marthaler D.G. (2017). Genome characterization of turkey rotavirus g strains from the united states identifies potential recombination events with human rotavirus b strains. J. Gen. Virol..

[6] Matthijnssens J., Otto P.H., Ciarlet M., Desselberger U., Van Ranst M., Johne R. (2012). Vp6-sequence-based cutoff values as a criterion for rotavirus species demarcation. Arch. Virol..

[7] Guo Y., Candelero-Rueda R.A., Saif L.J., Vlasova A.N. (2021). Infection of porcine small intestinal enteroids with human and pig rotavirus a strains reveals contrasting roles for histo-blood group antigens and terminal sialic acids. PLoS Pathog..

[8] Hu L., Sankaran B., Laucirica D.R., Patil K., Salmen W., Ferreon A.C.M., Tsoi P.S., Lasanajak Y., Smith D.F., Ramani S. (2018). Glycan recognition in globally dominant human rotaviruses. Nat. Commun..

[9] Estes M.K., Greenberg H.B., Fields B.N., Knipe D.M., Howley P.M. (2013). Fields Virology.

[10] Saif L.J., Jiang B. (1994). Nongroup a rotaviruses of humans and animals. Curr. Top. Microbiol. Immunol..

[11] Bridger J.C., Pedley S., McCrae M.A. (1986). Group c rotaviruses in humans. J. Clin. Microbiol..

[12] Marthaler D., Homwong N., Rossow K., Culhane M., Goyal S., Collins J., Matthijnssens J., Ciarlet M. (2014). Rapid detection and high occurrence of porcine rotavirus a, b, and c by rt-qpcr in diagnostic samples. J. Virol. Methods.

[13] Trovão N.S., Shepherd F.K., Herzberg K., Jarvis M.C., Lam H.C., Rovira A., Culhane M.R., Nelson M.I., Marthaler D.G. (2019). Evolution of rotavirus c in humans and several domestic animal species. Zoonoses Public Health.

[14] Vlasova A.N., Amimo J.O., Saif L.J. (2017). Porcine rotaviruses: Epidemiology, immune responses and control strategies. Viruses.

[15] Saif L.J., Bohl E.H., Theil K.W., Cross R.F., House J.A. (1980). Rotavirus-like, calicivirus-like, and 23-nm virus-like particles associated with diarrhea in young pigs. J. Clin. Microbiol..

[16] Kim Y., Chang K.O., Straw B., Saif L.J. (1999). Characterization of group c rotaviruses associated with diarrhea outbreaks in feeder pigs. J. Clin. Microbiol..

[17] Amimo J.O., Vlasova A.N., Saif L.J. (2013). Prevalence and genetic heterogeneity of porcine group c rotaviruses in nursing and weaned piglets in ohio, USA and identification of a potential new vp4 genotype. Vet. Microbiol..

[18] Chepngeno J., Diaz A., Paim F.C., Saif L.J., Vlasova A.N. (2019). Rotavirus c: Prevalence in suckling piglets and development of virus-like particles to assess the influence of maternal immunity on the disease development. Vet. Res..

[19] Wang Y., Porter E.P., Lu N., Zhu C., Noll L.W., Hamill V., Brown S.J., Palinski R.M., Bai J. (2021). Whole-genome classification of rotavirus c and genetic diversity of porcine strains in the USA. J. Gen. Virol..

[20] Moutelíková R., Prodělalová J., Dufková L. (2015). Diversity of vp7, vp4, vp6, nsp2, nsp4, and nsp5 genes of porcine rotavirus c: Phylogenetic analysis and description of potential new vp7, vp4, vp6, and nsp4 genotypes. Arch. Virol..

[21] Marthaler D., Rossow K., Culhane M., Collins J., Goyal S., Ciarlet M., Matthijnssens J. (2013). Identification, phylogenetic analysis and classification of porcine group c rotavirus vp7 sequences from the united states and canada. Virology.

[22] Terrett L.A., Saif L.J. (1987). Serial propagation of porcine group c rotavirus (pararotavirus) in primary porcine kidney cell cultures. J. Clin. Microbiol..

[23] Foulke-Abel J., In J., Kovbasnjuk O., Zachos N.C., Ettayebi K., Blutt S.E., Hyser J.M., Zeng X.L., Crawford S.E., Broughman J.R. (2014). Human enteroids as an ex-vivo model of host-pathogen interactions in the gastrointestinal tract. Exp. Biol. Med. (Maywood N.J.).

[24] Sato T., Stange D.E., Ferrante M., Vries R.G., Van Es J.H., Van den Brink S., Van Houdt W.J., Pronk A., Van Gorp J., Siersema P.D. (2011). Long-term expansion of epithelial organoids from human colon, adenoma, adenocarcinoma, and barrett’s epithelium. Gastroenterology.

[25] Sato T., Clevers H. (2013). Growing self-organizing mini-guts from a single intestinal stem cell: Mechanism and applications. Science.

[26] Middendorp S., Schneeberger K., Wiegerinck C.L., Mokry M., Akkerman R.D., van Wijngaarden S., Clevers H., Nieuwenhuis E.E. (2014). Adult stem cells in the small intestine are intrinsically programmed with their location-specific function. Stem Cells.

[27] Li L., Fu F., Guo S., Wang H., He X., Xue M., Yin L., Feng L., Liu P. (2019). Porcine intestinal enteroids: A new model for studying enteric coronavirus porcine epidemic diarrhea virus infection and the host innate response. J. Virol..

[28] Pearce S.C., Weber G.J., van Sambeek D.M., Soares J.W., Racicot K., Breault D.T. (2020). Intestinal enteroids recapitulate the effects of short-chain fatty acids on the intestinal epithelium. PLoS ONE.

[29] Saxena K., Blutt S.E., Ettayebi K., Zeng X.L., Broughman J.R., Crawford S.E., Karandikar U.C., Sastri N.P., Conner M.E., Opekun A.R. (2016). Human intestinal enteroids: A new model to study human rotavirus infection, host restriction, and pathophysiology. J. Virol..

[30] Ettayebi K., Crawford S.E., Murakami K., Broughman J.R., Karandikar U., Tenge V.R., Neill F.H., Blutt S.E., Zeng X.L., Qu L. (2016). Replication of human noroviruses in stem cell-derived human enteroids. Science.

[31] Zang R., Gomez Castro M.F., McCune B.T., Zeng Q., Rothlauf P.W., Sonnek N.M., Liu Z., Brulois K.F., Wang X., Greenberg H.B. (2020). Tmprss2 and tmprss4 promote sars-cov-2 infection of human small intestinal enterocytes. Sci. Immunol..

[32] Zhou J., Li C., Liu X., Chiu M.C., Zhao X., Wang D., Wei Y., Lee A., Zhang A.J., Chu H. (2020). Infection of bat and human intestinal organoids by sars-cov-2. Nat. Med..

[33] Luo H., Zheng J., Chen Y., Wang T., Zhang Z., Shan Y., Xu J., Yue M., Fang W., Li X. (2020). Utility evaluation of porcine enteroids as pdcov infection model in vitro. Front. Microbiol..

[34] Zhang D., Tan M., Zhong W., Xia M., Huang P., Jiang X. (2017). Human intestinal organoids express histo-blood group antigens, bind norovirus vlps, and support limited norovirus replication. Sci. Rep..

[35] Svensson L. (1992). Group c rotavirus requires sialic acid for erythrocyte and cell receptor binding. J. Virol..

[36] Sun X., Wang L., Qi J., Li D., Wang M., Cong X., Peng R., Chai W., Zhang Q., Wang H. (2018). Human group c rotavirus vp8*s recognize type a histo-blood group antigens as ligands. J. Virol..

[37] Zhao D., Liu Y., Huang P., Xia M., Li W., Tan M., Zhang X., Jiang X. (2020). Histo-blood group antigens as divergent factors of groups a and c rotaviruses circulating in humans and different animal species. Emerg. Microbes Infect..

[38] Park J.H., Lee K.W., Chung I.S. (1998). Improved propagation of human rotavirus from cell cultures of rhesus monkey kidney cells using medium supplemented with deae-dextran, dimethyl sulfoxide and cholesterol. Biotechnol. Tech..

[39] Cui J., Fu X., Xie J., Gao M., Hong M., Chen Y., Su S., Li S. (2014). Critical role of cellular cholesterol in bovine rotavirus infection. Virol. J..

[40] Bégin M.E. (1980). Enhanced production of infectious rotavirus in bsc-1 cell cultures by various factors in the presence of absence of trypsin. J. Gen. Virol..

[41] Chepngeno J., Takanashi S., Diaz A., Michael H., Paim F.C., Rahe M.C., Hayes J.R., Baker C., Marthaler D., Saif L.J. (2020). Comparative sequence analysis of historic and current porcine rotavirus c strains and their pathogenesis in 3-day-old and 3-week-old piglets. Front. Microbiol..

[42] Saif L.J., Terrett L.A., Miller K.L., Cross R.F. (1988). Serial propagation of porcine group c rotavirus (pararotavirus) in a continuous cell line and characterization of the passaged virus. J. Clin. Microbiol..

[43] Van der Hee B., Loonen L.M.P., Taverne N., Taverne-Thiele J.J., Smidt H., Wells J.M. (2018). Optimized procedures for generating an enhanced, near physiological 2d culture system from porcine intestinal organoids. Stem Cell Res..

[44] Barbe L., Le Moullac-Vaidye B., Echasserieau K., Bernardeau K., Carton T., Bovin N., Nordgren J., Svensson L., Ruvoen-Clouet N., Le Pendu J. (2018). Histo-blood group antigen-binding specificities of human rotaviruses are associated with gastroenteritis but not with in vitro infection. Sci. Rep..

[45] Okeley N.M., Alley S.C., Anderson M.E., Boursalian T.E., Burke P.J., Emmerton K.M., Jeffrey S.C., Klussman K., Law C.-L., Sussman D. (2013). Development of orally active inhibitors of protein and cellular fucosylation. Proc. Natl. Acad. Sci. USA.

[46] Zhang X., Chen F., Petrella A., Chacón-Huete F., Covone J., Tsai T.-W., Yu C.-C., Forgione P., Kwan D.H. (2019). A high-throughput glycosyltransferase inhibition assay for identifying molecules targeting fucosylation in cancer cell-surface modification. ACS Chem. Biol..

[47] Nguyen D., Choi H., Jo H., Kim J.H., Dirisala V., Lee K.T., Kim T.H., Park K.K., Seo K., Park C. (2011). Molecular characterization of the human abo blood group orthologus system in pigs. Anim. Genet..

[48] Lu X., Xiong Y., Silver J. (2002). Asymmetric requirement for cholesterol in receptor-bearing but not envelope-bearing membranes for fusion mediated by ecotropic murine leukemia virus. J. Virol..

[49] Anderson H.A., Chen Y., Norkin L.C. (1996). Bound simian virus 40 translocates to caveolin-enriched membrane domains, and its entry is inhibited by drugs that selectively disrupt caveolae. Mol. Biol. Cell.

[50] Huang L., Zhang Y.-P., Yu Y.-L., Sun M.-X., Li C., Chen P.-Y., Mao X. (2011). Role of lipid rafts in porcine reproductive and respiratory syndrome virus infection in marc-145 cells. Biochem. Biophys. Res. Commun..

[51] Yin J., Glende J., Schwegmann-Wessels C., Enjuanes L., Herrler G., Ren X. (2010). Cholesterol is important for a post-adsorption step in the entry process of transmissible gastroenteritis virus. Antivir. Res..

[52] Zhu L., Ding X., Tao J., Wang J., Zhao X., Zhu G. (2010). Critical role of cholesterol in bovine herpesvirus type 1 infection of mdbk cells. Vet. Microbiol..

[53] Thorne C.A., Chen I.W., Sanman L.E., Cobb M.H., Wu L.F., Altschuler S.J. (2018). Enteroid monolayers reveal an autonomous wnt and bmp circuit controlling intestinal epithelial growth and organization. Dev. Cell.

[54] Almeida P.R., Lorenzetti E., Cruz R.S., Watanabe T.T., Zlotowski P., Alfieri A.A., Driemeier D. (2018). Diarrhea caused by rotavirus a, b, and c in suckling piglets from southern brazil: Molecular detection and histologic and immunohistochemical characterization. J. Vet. Diagn. Investig..

[55] Tsunemitsu H., Saif L.J., Jiang B.M., Shimizu M., Hiro M., Yamaguchi H., Ishiyama T., Hirai T. (1991). Isolation, characterization, and serial propagation of a bovine group c rotavirus in a monkey kidney cell line (ma104). J. Clin. Microbiol..

[56] Welter M.W., Welter C.J., Chambers D.M., Svensson L. (1991). Adaptation and serial passage of porcine group c rotavirus in st-cells, an established diploid swine testicular cell line. Arch. Virol..

[57] Hellman S. (2021). Generation of equine enteroids and enteroid-derived 2d monolayers that are responsive to microbial mimics. Vet. Res..

[58] Hu L., Crawford S.E., Czako R., Cortes-Penfield N.W., Smith D.F., Le Pendu J., Estes M.K., Prasad B.V. (2012). Cell attachment protein vp8* of a human rotavirus specifically interacts with a-type histo-blood group antigen. Nature.

[59] Liu Y., Huang P., Tan M., Liu Y., Biesiada J., Meller J., Castello A.A., Jiang B., Jiang X. (2012). Rotavirus vp8*: Phylogeny, host range, and interaction with histo-blood group antigens. J. Virol..

[60] Marionneau S., Cailleau-Thomas A., Rocher J., Le Moullac-Vaidye B., Ruvoen N., Clement M., Le Pendu J. (2001). ABH and lewis histo-blood group antigens, a model for the meaning of oligosaccharide diversity in the face of a changing world. Biochimie.

[61] Heggelund J.E., Varrot A., Imberty A., Krengel U. (2017). Histo-blood group antigens as mediators of infections. Curr. Opin. Struct. Biol..

[62] Jiang X., Liu Y., Tan M. (2017). Histo-blood group antigens as receptors for rotavirus, new understanding on rotavirus epidemiology and vaccine strategy. Emerg. Microbes Infect..

[63] Ciarlet M., Ludert J.E., Iturriza-Gómara M., Liprandi F., Gray J.J., Desselberger U., Estes M.K. (2002). Initial interaction of rotavirus strains with n-acetylneuraminic (sialic) acid residues on the cell surface correlates with vp4 genotype, not species of origin. J. Virol..

[64] Ruggeri F.M., Greenberg H.B. (1991). Antibodies to the trypsin cleavage peptide vp8 neutralize rotavirus by inhibiting binding of virions to target cells in culture. J. Virol..

[65] Ciarlet M., Estes M.K. (1999). Human and most animal rotavirus strains do not require the presence of sialic acid on the cell surface for efficient infectivity. J. Gen. Virol..

[66] Kuhlenschmidt T.B., Hanafin W.P., Gelberg H.B., Kuhlenschmidt M.S. (1999). Sialic acid dependence and independence of group a rotaviruses. Adv. Exp. Med. Biol..

[67] Delorme C., Brüssow H., Sidoti J., Roche N., Karlsson K.A., Neeser J.R., Teneberg S. (2001). Glycosphingolipid binding specificities of rotavirus: Identification of a sialic acid-binding epitope. J. Virol..

[68] Haselhorst T., Fleming F.E., Dyason J.C., Hartnell R.D., Yu X., Holloway G., Santegoets K., Kiefel M.J., Blanchard H., Coulson B.S. (2009). Sialic acid dependence in rotavirus host cell invasion. Nat. Chem. Biol..

[69] Guo Y. (2021). Porcine Intestinal Enteroids: A Novel Model to Study Host Glycan-Rotavirus Interaction.

[70] Loh P.C., Hashiro G.M., Yau J.T. (1977). Effect of polycations on the early stages of reovirus infection. Microbios.

[71] Simons K., Ikonen E. (1997). Functional rafts in cell membranes. Nature.

[72] Simons K., van Meer G. (1988). Lipid sorting in epithelial cells. Biochemistry.

[73] Parton R.G., Hancock J.F. (2004). Lipid rafts and plasma membrane microorganization: Insights from ras. Trends Cell Biol..

[74] Nayak D.P., Barman S. (2002). Role of lipid rafts in virus assembly and budding. Adv. Virus Res..

[75] Wang Y., Zhang Y., Zhang C., Hu M., Yan Q., Zhao H., Zhang X., Wu Y. (2020). Cholesterol-rich lipid rafts in the cellular membrane play an essential role in avian reovirus replication. Front. Microbiol..

[76] Veit M., Thaa B. (2011). Association of influenza virus proteins with membrane rafts. Adv. Virol..

