# Supplementary material for: Rotavirus C Replication in Porcine Intestinal Enteroids Reveals Roles for Cellular Cholesterol and Sialic Acids
Source: Viruses. 2022 Aug 20;14(8):1825. doi: 10.3390/v14081825 (PMC9416568; doi:10.3390/v14081825)
Supplement: Supplementary file 1 [file viruses-14-01825-s001.zip › viruses-1841224-supplementary.pdf]

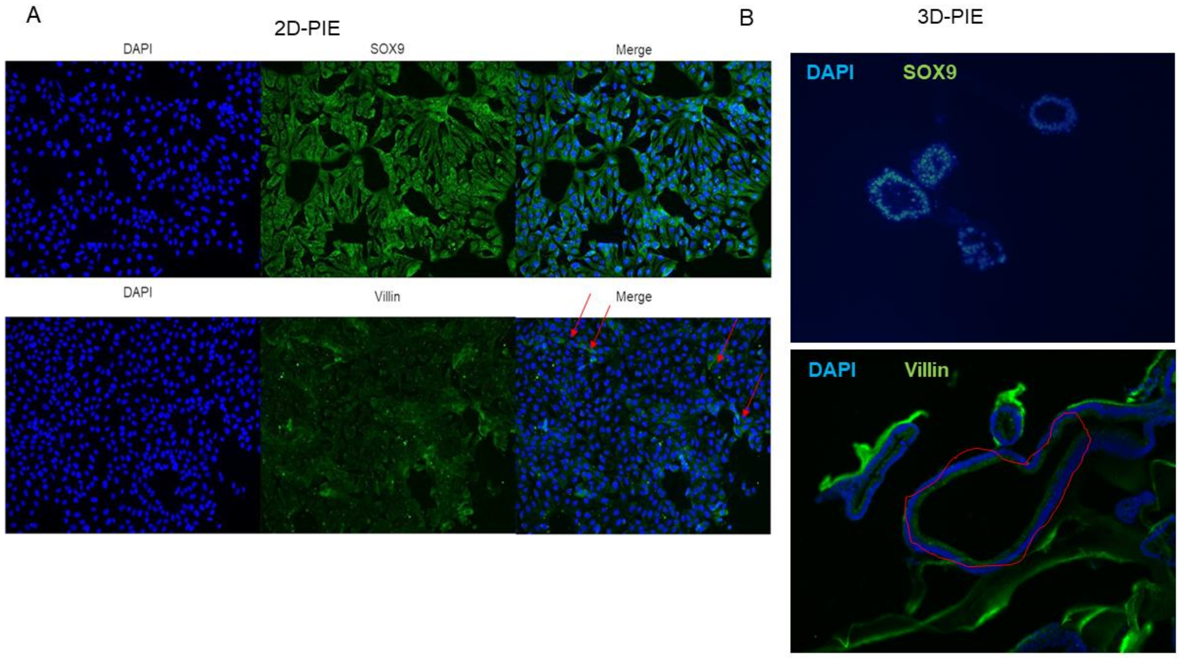

Figure S1. Marker expression of 2D monolayer. IF analysis of expression of different proteins on the 2D monolayer and 3D-PIEs. A: SOX9 (upper panel) and villin (lower panel) expression on the (**A**) 2D monolayer of PIEs and (**B**) 3D-PIEs. Anti-SOX9 or anti-villin and DAPI staining are shown in green and blue colors, respectively. Red arrows and red lines show villin + cells in 2D and 3D-PIE, respectively.
